# Non-Analgesic Symptomatic or Disease—Modifying Potential of TRPA1

**DOI:** 10.3390/medsci7100099

**Published:** 2019-09-23

**Authors:** Stefan Heber, Michael J.M. Fischer

**Affiliations:** Center for Physiology and Pharmacology, Medical University of Vienna, Vienna 1090, Austria; stefan.heber@meduniwien.ac.at

**Keywords:** therapy, pharmacology, pain, itch, drug development, ion channel, clinical study

## Abstract

TRPA1, a versatile ion channel of the Transient Receptor Potential (TRP) channel family, detects a large variety of chemicals and can contribute to signal processing of other stimuli, e.g., due to its sensitivity to cytosolic calcium elevation or phosphoinositolphosphate modulation. At first, TRPA1 was found on sensory neurons, where it can act as a sensor for potential or actual tissue damage that ultimately may elicit pain or itch as warning symptoms. This review provides an update regarding the analgesic and antipruritic potential of TRPA1 modulation and the respective clinical trials. Furthermore, TRPA1 has been found in an increasing amount of other cell types. Therefore, the main focus of the review is to discuss the non-analgesic and particularly the disease-modifying potential of TRPA1. This includes diseases of the respiratory system, cancer, ischemia, allergy, diabetes, and the gastrointestinal system. The involvement of TRPA1 in the respective pathophysiological cascades is so far mainly based on pre-clinical data.

## 1. Overview

Since its cloning [1], description of its role in the sensory system [2], phenotyping of the knockout animals [3], and determination of the majority of the structure [4], Transient receptor potential cation channel subfamily A member 1 (TRPA1) has been the topic of several reviews. These were more general [5] or more topical, e.g., with a molecular perspective [6]. In addition, we refer to previous reviews dedicated to druggability [7], or focused on analgesic potential [8,9]. The present review considers targeting TRPA1 in clinical studies for a specific disease and lists evidence for TRPA1 as a potential therapeutic target in diseases not yet addressed in clinical studies, particularly considering non-analgesic potential. An important point is the therapeutic potential of pathways which involve TRPA1 downstream, e.g., due to its calcium- and Phosphatidylinositol-4,5-bisphosphat-dependence. Therefore, other upstream targets activating G_s_ or G_q_-coupled G protein-coupled receptors might also have a secondary, so far potentially overlooked TRPA1 component in their pathophysiology [7]. Finally, the review discusses potential side effects of TRPA1 modulation, which could prevent, or at least largely limit, its therapeutic use.

## 2. TRPA1-Related Substances

A large fraction of the major pharmaceutical players had or have development programs for TRPA1, and the compilation below represents only the visible tip of the iceberg. In 2007, HC–030031 developed by Hydra Biosciences was the first TRPA1 antagonist [10]. In the same year, Amgen published trichloro(sulfanyl)ethyl benzamides as potent TRPA1 antagonists, which were more than 10 times more potent in human compared to rat TRPA1 [11]. Species selectivity of TRPA1 could be at least partially attributed to amino acids of transmembrane domain 5 and the pore loop [12]. AP–18, reported in 2007 [13], has a similar potency to HC–030031. In 2009, Abbott disclosed the potent oxime derivative A–967079 (patent WO/2009/089082). The structure of A–967079 is similar to AP–18, but is more species-selective, providing a substantially improved IC50 of 67 nM on human TRPA1 (and 289 nM on rat TRPA1). Soon after becoming commercially available in 2012, A–967079 became the gold standard for experimental TRPA1 inhibition. In 2010, Glenmark Pharmaceuticals reported GRC17536, which has been pursued in clinical trials. Further substances which were advanced to phase I trials include CB–625 and ODM–108; for details, we refer to a recent review [8]. The latter three substances are currently not commercially available.

Finally, TRPA1 agonists should be mentioned, as the availability and specificity of the tools also affect the conclusions which can be drawn. The tools to activate TRPA1 have been rather poor for a long time, and substances with a limited range of specificity have been used at concentrations exceeding this range. Compared to e.g., allyl isothiocyanate (AITC), cinnamaldehyde, acrolein, or carvacrol, newer substances like JT010 and PF–4840154 allow potent and selective agonism [14,15]. TRPA1 activation reliably generates pain in humans, and models include AITC, cinnamaldehyde, carvacrol, and JT010 [16,17,18,19]. With a specific TRPA1 stimulation, sensations were not different from those generated by capsaicin, with the subjects reporting a primarily burning pain. Importantly, subjects stated that they did not experience cold sensations [20].

So far, no TRPA1 antagonists have been validated for therapeutic use, indicating unforeseen events in the development or limited efficacy in the chosen models, which has probably led to termination of TRPA1-focussed drug development by many pharmaceutical companies. However, the soaring output of TRPA1-related research provided an unexpectedly wide diversification of roles for TRPA1, and investigations for potential applications for pain and non-pain targets are underway. 

## 3. Symptomatic Therapy—Analgesic Potential

The potential of TRPA1-related drugs, primarily of TRPA1 antagonists, for the treatment of pain has been discussed in several reviews. Therefore, the aim of this paragraph is not to cover this extensively, but to provide an overview with reference to the respective literature.

It should be mentioned that TRPA1 expression in the nervous system dominated the literature before TRPA1 had stepwise been found in other tissues. The phenotype of TRPA1 knockout mice [3,21] showed mixed results regarding mechanical and cold sensitivity, but without doubt, a deficit for irritants and a role in inflammatory conditions. This explains an initial focus on pain, or at least disease processes where the aim of targeting TRPA1 was to control pain. The respective disease areas seem diverse when listed, and include postoperative pain, osteoarthritis, pain due to bacterial inflammation, gastrointestinal inflammatory pain, migraine, and also neuropathic pain, including diabetic neuropathy, peripheral traumatic neuropathy and chemotherapy-induced neuropathy [8]. However, this could also be viewed from a more pathophysiological perspective, considering conditions with oxidative stress and inflammatory sensitization, all of which activate or sensitize TRPA1, or lead to its overexpression.

The majority of the work is based on animal experiments. Clinical studies to treat pain with a TRPA1 antagonist are rather limited, which might be the result of a translational barrier. The larger genetic distance between humans and mice compared to other ion channels, e.g., TRPV1, might explain the pronounced species differences, which have been described for TRPA1 [6]. This applies not only to evolutionary distant but also to closer species, and has been used to identify molecular determinants as well as for drug development [22,23,24,25,26,27].

ClinicalTrial.gov lists 9 entries for TRPA1, of which three are completed. The pain-related trials are discussed here, and the non-pain related trials below. Glenmark Pharmaceuticals tested GRC17536 in a phase 2a clinical trial for diabetic neuropathy (NCT01726413, EudraCT Number 2012-002320-33). The study failed its primary endpoint 24-h average pain intensity. However, a post-hoc exploratory subgroup analysis suggested that GRC17536 might potentially be effective in “an irritable nociceptor subgroup” of patients, in which peripherally driven symptoms can be assumed (patent WO/2016/042501). This hypothesis, however, has never been tested.

In trial NCT02653703, menthol, which is an agonist at TRPM8 and human TRPA1 [12], could reduce trans-cinnamaldehyde-induced hyperalgesia and neurogenic inflammation [18]. A study regarding the involvement of TRPA1 in the relationship between cold exposure and chemotherapy-induced neuropathy has not been completed yet (NCT03329131), which also applies to a trial which investigates genetic variations in platinum-based chemotherapy-induced neuropathy (NCT03252834). Contribution of TRPA1 to heritable pain syndromes is investigated in NCT02696746 by correlating TRPA1 mutations with symptoms of pain in an observational study. A regulation of TRPA1 expression by intraoperative ketamine application is considered as a potential mechanism in NCT02729805. In addition, there are several drug candidates terminated in Phase 1, for which a favorable preclinical profile can be assumed, but there is obviously little incentive to share insights in a competitive market. This includes e.g., CB–625 by Cubist Pharmaceuticals or ODM–108 by Orion Pharma. Nevertheless, it has to be mentioned that, despite investigation for quite some years, there is astonishingly limited evidence from clinical pain trials for efficacy of TRPA1 inhibition.

Headaches can be differentiated into many subcategories, but many of these share the pathophysiology of pain and inflammation with other areas of the body. The majority of the migraine triggers can generate oxidative stress [28]. These triggers include various pathomechanisms, ranging from neuronal overactivation in an excitotoxic manner to downstream or direct targeting of mitochondrial function, or inhibition of scavenging pathways for cellular stress. A common denominator is the generation of reactive molecular species, an umbrella term to summarize many molecular entities, including reactive oxygen, nitrogen, and carbonyl species (ROS, RNS, RCS) and combinations thereof, but also endogenous lipid mediators. TRPA1 activation by these mediators can release further headache-provoking substances [29] and there is an epidemiological association between environmental exposure to TRPA1 activators and migraine [30,31]. Migraine-related involvement of TRPA1 has been addressed in a topical review [32].

In hindlimb ischemia as a model for peripheral arterial disease, spontaneous pain-related behaviour during reperfusion was reduced in TRPA1 knockouts and by TRPA1 inhibition [33]. TRPA1 inhibition might also help patients to cope with pathological response to cold challenge [34], which occurs e.g., in Raynaud syndrome patients. For both diseases with temporary insufficient perfusion, TRPA1 inhibition is assumed to be symptomatic, but not to alter the course of the disease.

## 4. Symptomatic Therapy—Antipruritic Potential

Itch and pain are distinct sensations. However, the predominant view is that there are no neurons exclusively sensing pruritogens. Neuronal activation generates pain and itch with at least partially overlapping neuronal subpopulations [35,36,37]. There is ample evidence that TRPA1 contributes to itch. Acetone–ether–water mixture causes scratching, which is absent in TRPA1 knockouts [38]. TRPA1 is involved in neuronal excitation, causing itch by direct TRPA1 activation [39]. Similarly, the TLR7 agonist imiquimod can directly activate TRPA1, causing itch in mice [40], the latter being investigated in humans in trial NCT03943407. TRPA1 involvement can also occur downstream, particularly in models which have a defined receptor target associated with itch, e.g., using SLIGRL to activate MrgprC11 [41], chloroquine to activate Mrgpra3/X1 [42], serotonin to activate HTR7 [43], TSLP to activate the TSLP receptor [44], and IL31 to activate the IL31 receptor [45]. These diverse mediators converge on common pathways as modulating G protein-coupled receptors, and TRPA1 has been shown to be essential in these pathways [46,47,48,49].

Importantly, TRPA1 contributes to maintaining skin inflammation, and inhibition might therefore be disease-modifying, reducing skin swelling, leukocyte infiltration, and transepidermal water loss. [38,50,51]. Atopic dermatitis is a burdensome allergic disease with therapy-resistant chronic itch as a hallmark. In an oxazolone-based animal model of chronic dermatitis, TRPA1 inhibition could reduce scratching [47]. Scratching induced by IL-13 or IL-31 in atopic dermatitis models was reduced in TRPA1-deficient mice or by TRPA1 inhibition [45,50]. TRPA1 activation in mast cells might add to the disease, but the data do not allow to conclude that TRPA1 inhibition would be disease-modifying, which would qualify for the following chapter.

## 5. Potential Disease—Modification through TRPA1 Modulation

The general role of TRPA1 in non-neuronal tissue is similar to the one in neuronal tissue: it senses reactive molecules [52,53]. Thus, there are two principal mechanisms by which TRPA1 activation or inhibition could modify diseases. First, TRPA1-expressing cells could be influenced directly. Alternatively, the sensory neurons supplying a tissue could modulate inflammatory processes by the release of neuropeptides, which might act directly on parenchymal cells or by means of chemoattraction or immunomodulation. Both mechanisms might be disease-modifying. Consequently, TRPA1 expression by non-neuronal cells of a tissue or organ should not be considered a prerequisite for the possibility of disease modification via TRPA1, as almost all tissues are innervated.

As example for disease modification might serve a disruption of the inflammatory cascade by prolonged TRPA1 inhibition using HC–030031, with reduced inflammation, shown by neutrophil immigration and oxidative stress [54].

### 5.1. Respiratory Tract and Lung

In the respiratory tract, both supplying sensory neurons and non-neuronal cells express TRPA1, e.g., epithelial cells, smooth muscle cells, and invading immune cells [55,56]. Exposure to a TRPA1 agonist causes coughing in animals and humans [57,58,59]. Therefore, the normal function might be to protect the airways by evoking cough upon exposure to respiratory irritants. TRPA1 inhibition could be a symptomatic treatment for chronic cough, but on the other hand, might increase the uptake of harmful substances into the airways by reducing an important protective reflex. TRPA1 in the airways has also a major role in the generation of other nocifensive responses, such as mucus production, adjustment of bronchus diameter, and inflammation [60]. These responses are not pathological, but rather a physiological response to irritation. However, animal studies with genetic ablation or pharmacological inhibition of TRPA1 suggest that the channel is causally involved in airway inflammation under pathological conditions, such as asthma or chronic obstructive pulmonary disease [55,61,62]. The role of TRPA1 is also supported by single nucleotide polymorphisms in humans, which are associated with an increased risk for diagnosed asthma [63].

Furthermore, animal studies suggest that in the case of inflamed airways, TRPA1 might add insult to injury when endogenous inflammatory mediators prostaglandin E2 and bradykinin activate TRPA1 and further amplify the neurogenic contribution to inflammation [64]. Interestingly, both constriction and dilation of airways were reported in response to TRPA1 activation. While isolated guinea pig bronchi were found to constrict [65], isolated mouse tracheae were reported to dilate [66]. These opposing results might be due to different airway sections or the respective species.

Overall, bronchoconstriction and airway hyperreactivity could lead to more mucus retention and inflammation, and therefore might not only aggravate symptoms, but also progress the disease [67]. Also, an exaggerated inflammatory response might switch TRPA1 activation from protective to harmful, with all detrimental effects of chronic inflammation, including poorly reversible fibrosis and reduced lung compliance. Therapeutic potential for respiratory disorders has been addressed in a topical review [68].

Results from the registered human trials (NCT02591550, NCT02039999) on TRPA1 contribution to coughing have not been reported yet. A combined phase I/IIa study to evaluate inhaled GRC17536 in healthy volunteers and asthma patients has also been registered, but not been published (NHS, IRAS ID 110883). Considering substantial species differences, the role of TRPA1 in human airway inflammation remains unknown. Randomized controlled trials are required to establish whether TRPA1 might serve as a drug target to modify disease progression in humans.

### 5.2. Cancer

The role of TRP channels, particularly in lung cancer, has been discussed [56]. Small cell lung cancer cell lines were found to functionally express TRPA1 and activation of TRPA1 prevented apoptosis and led to increased cell survival [69], suggesting a potential role in tumour progression and a disease-modifying effect of chronic TRPA1 inhibition. Similarly, in breast cancer cells and spheroids, it was shown that TRPA1 sensing of cellular redox stress can activate anti-apoptotic programs. This led to increased oxidative stress tolerance, and TRPA1 inhibition could reduce tumour growth and enhance chemotherapy sensitivity in xenograft models [70]. TRPA1 expression has been shown to be highest in the mentioned tumour types, and it remains to be clarified whether the described mechanisms apply to other tumours.

### 5.3. Ischemia

Temporary ischemia in the heart or in the brain often has detrimental results for the subject. For these major disease entities, but also for peripheral artery disease, pre-clinical studies suggest the role of TRPA1.

In cardiac ischemia, the majority of damage occurs during reperfusion injury, when a high level of oxidative stress destroys cardiac tissue. Additional calcium, admitted through TRPA1 as a sensor for oxidative stress, might aggravate tissue injury [71]. In this study, TRPA1 deficient mice had smaller infarctions, with similar tissue at risk. Inhibition of TRPA1 also reduced damage in isolated cardiomyocytes. This suggests that TRPA1 might be involved in myocardial damage in response to ischemia, however, clinical studies have not been performed.

In the brain, TRPA1 is found on oligodendrocytes. Ischemia was found to cause demyelination, and the identified pathway includes acidosis, TRPA1 activation, and increased calcium load [72]. Ischemic damage was less pronounced in TRPA1 knockouts and could be inhibited by several TRPA1 antagonists. However, also neuroprotective effects of TRPA1 in cerebral ischemia need to be considered. TRPA1 expressed by endothelial cells of rodent cerebral and cerebellar pial arteries can induce cerebral artery dilation [73]. In a model of ischemic stroke, endothelial cell specific TRPA1-knockout animals exhibited larger infarct sizes, supporting the view that TRPA1 activation by endogenous agonists generated during ischemia can have protective effects as well [74]. Thus, TRPA1 activation in human ischemia might turn out to be a two-edged sword, where both favourable and unfavourable effects result from TRPA1 modulation. Before clinical trials can reasonably be performed, a more thorough understanding of TRPA1 contribution in cardiac physiology and pathophysiology needs to be established in pre-clinical studies. In summary, the net importance of TRPA1 is unclear, and might even depend on level, extent and duration of ischemia.

### 5.4. Allergic Conjunctivitis

Recently, it was suggested that TRPA1 might also be involved in allergic conjunctivitis [75]. Compared to wild-type animals, TRPA1 deficient mice show fewer symptoms in a model of allergic conjunctivitis [76]. The TRPA1 receptor levels at the cell surface were shown to be increased by nociceptive stimuli and inflammatory conditions [77]. Neuropeptide release in response to TRPA1 activation can stimulate other players in the inflammatory process, generating a vicious cycle which might be interrupted by TRPA1 inhibition. However, the principal causative agent is an antigen binding to IgE on mast cells, causing their degranulation. Therefore, it is unclear whether TRPA1 inhibition is sufficient to modify the disease.

### 5.5. Diabetes

TRPA1 was found to be expressed in rat pancreatic beta-cells, where it seems to act synergistically with K_ATP_ channels leading to insulin secretion [78]. This might imply that systemic TRPA1 agonists decrease blood glucose at first, but also contribute to beta-cell dysfunction in the long run based on beta-cell exhaustion [79]. What might also be relevant is that glibenclamide, an anti-diabetic drug that increases insulin secretion from beta-cells, activates TRPA1 [80]. However, the role of TRPA1 agonism in insulin secretion needs further validation. Cinnamaldehyde improves glucose tolerance, but without change in plasma insulin levels [81]. In analogy, a reduced oral glucose tolerance in presence of TRPA1 antagonism should be a major concern, but is not supported by observations in other laboratories (personal communication).

Considering disease progression, it is important to at least retard the sequelae of chronically increased blood glucose. Methylglyoxal, a metabolite that occurs as a side-product of glycolysis [82], is increased in diabetic populations [83]. This is thought to be detrimental to neuronal function and might contribute to positive symptoms via activation of TRPA1 [84] as well as to the negative symptoms due to the loss of nerve fibres. Concerning this, chronic inhibition of TRPA1 might be expected to reduce neuropathy in diabetes. However, effects of human short-term exposure should be explored before this hypothesis is addressed.

### 5.6. Gastrointestinal Tract and Skin

In inflammatory bowel disease, the evidence regarding TRPA1 involvement is far from unequivocal. TRPA1 expression seems to be altered in mucosal tissue, however, whether TRPA1 modulation exacerbates or ameliorates disease activity depends on the used animal model [85]. The neuropeptides Substance P and CGRP seem to be involved, which contribute to intestinal inflammation [86]. Reducing neuropeptide release by TRPA1 inhibition could reduce inflammation and support intestinal barrier integrity, which is likely to be disease-modifying. A reduction of intestinal permeability by oral cinnamaldehyde has been shown in porcine epithelial cells [87]. The only clinical study was performed in healthy volunteers and included cinnamaldehyde as a TRPA1 agonist and capsaicin as a TRPV1 agonist according to registration (NCT01667523). The primary endpoint was intestinal permeability as measured by plasma concentrations of GLP–1 and PYY, but in contrast to trial registration, only data regarding capsaicin were published [88]. Thus, the sample size calculation was not performed based on the provided primary outcome variable, which weakens the results. Taken together, a TRPA1-based modulation of inflammatory bowel disease could be therapeutic, but clinical trials are still missing. In experimental pancreatitis, early inhibition of TRPA1 reduced progression to chronic inflammation [89]. Regarding pharmacological exposure of a surface, the skin should also be considered. TRPA1 can facilitate [51,90] but also counteract skin inflammation [91], depending on the type and pathophysiology of the inflammation. Topical targeting of TRPA1 appears as an option. Systematic investigation of e.g., monoterpenoids, which have a high toxicity in many insects but a low toxicity in humans, has shown several TRPA1 inhibitors [92]. Within these, several have anti-inflammatory potential [93].

## 6. Adverse Effects

Currently, there are few undesired adverse effects known, and in particular, no class effects. While dietary supplementation with cinnamon left ambiguous results [94,95], administration of cinnamaldehyde or AITC was shown to prevent weight gain and hepatic steatosis caused by high fat diet [96,97]. Further improvements included regulation of anorexigenic markers, blood glucose and HbA1c levels, and insulin resistance. Such benefits of TRPA1 agonism might be considered as indirect evidence of the adverse effects of chronic TRPA1 antagonism [98]. However, it seems too speculative to draw any conclusion whether this could restrict or preclude TRPA1 antagonists from therapeutic use.

## 7. Conclusions

After years of development by many companies, sufficiently developed substances can be assumed. The initial focus was on pain-related targets, and these remain promising. However, other diseases should also be considered. So far, these have only marginally been explored in clinical studies.
